# The ototoxic effect of intratympanic terbinafine applied in the middle ear of rats

**DOI:** 10.1186/1916-0216-42-13

**Published:** 2013-02-04

**Authors:** Mustafa Sagit, Mehmet Akıf Somdas, Ferhat Korkmaz, Alper Akcadag

**Affiliations:** 1 Department of ENT, Kayseri Training and Research Hospital, Sanayi Mah. Atatürk Bulvarı Hastane Cad. No: 78, Kayseri, 38010, Turkey; 2 Kayseri Training and Research Hospital, Subdepartment of Audiology, Kayseri, Turkey

**Keywords:** Terbinafine, Ototoxicity, Intratympanic, Otomycosis, Distortion product otoacoustic emission, Auditory brain stem responses

## Abstract

**Background:**

Otomycosis is defined as an infection of the external ear canal with fungal agents. The treatment of the disease is cleansing and drying of the external ear canal, identification and treatment of any predisposing factors and application of topical antifungal agents. Terbinafine is used as an antifungal agent to treat otomycosis. We proposed to investigate the probable ototoxic effect of terbinafine solution on auditory brain stem response (ABR) and distortion product otoacoustic emission (DPOAE) when applied intratympanically in the middle ear of rats.

**Methods:**

The experiment was performed on 30 female Wistar albino rats. Thirty animals were divided into three groups of 10 animals each. 1% terbinafine solution was administered to the first group (group T). The second group (group G) was administered 40 mg/ml gentamicin solution (ototoxic control). The third group (group S) was administered saline solution (negative control). Baseline DPOAE measurements and ABR testing from the left ears were obtained from the animals in all groups under general anesthesia. Ear solutions were applied in the middle ear intratympanically with a dental needle. Treatment was initiated after baseline measurements and repeated once every two days for fifteen days.

**Results:**

Pre and post-treatment DPOAE responses for all tested frequencies of group T and Group S showed no statistically significant difference. However, the group G demonstrated a significant change in ABR thresholds and DPOAE responses.

**Conclusions:**

Terbinafine solution is a broad spectrum antifungal agent effective in the treatment of otomycosis. The present study demonstrated that its direct administration in the middle ear of rats does not affect inner ear function as measured by ABR and DPOAE responses.

## Background

Otomycosis is defined as an infection of the external ear canal with fungal agents. This includes not only the external ear canal but also open mastoid cavities and the middle ear [1]. The most common etiological agents are Candida and Aspergillus [2-4]. Infection is mainly characterized by pruritus, otalgia, tinnitus and hearing loss [1,4]. The most important point for the treatment of the disease is cleansing and drying of the external ear canal, identification and treatment of any predisposing factors and application of topical antifungal agents [5,6].

Terbinafine, which is from the allylamine group, is used as an antifungal agent. There are oral and topical formulations to treat superficial fungal infections. Although terbinafine is frequently used in the treatment of dermatophyte infections, it’s in vitro effects against Candida, Aspergillus, and other pathogenic fungi agents has been demonstrated [7,8]. Terbinafine has also shown fungicidal activity in vitro against Aspergillus species that lead to otomycosis [9].

The potential ototoxicity of antifungal drugs has been reported in different experimental animal studies. Clotrimazole, miconazole, nystatin and tolnaftate have shown no ototoxic effects when used as topical applications in guinea pigs, however gentian violet has shown ototoxic effects [5]. Cresylate and VoSol (hydrocortisone and acetic acid, nonaqueous 2%) have also shown ototoxicity in experimental animal studies [10]. Ciclopirox has shown no ototoxicity when administered in the middle ear of the guinea pig [11]. We found only one recently published study assessing ototoxic effect of terbinafine in the literature [12].

In our study, we aimed to assess the probable ototoxic effect of terbinafine solutions on ABR and DPOAE when applied intratympanically in the middle ear of rats.

## Methods

### Animals and groups

This study was approved by the Ethical Committee on Animal Research of Erciyes University, Kayseri, Turkey. The study was performed at the Experimental Animals Studies Laboratory of Erciyes University. In this study, 30 female Wistar albino rats which approximately weighing 200-220 g and 20 weeks of age were used. The animals were kept in ordinary cages in a temperature controlled room that maintained a 12-hour light/dark cycle. The animals were supplied with free reach to food and water. At the beginning of the study, we used an operating ear microscope to examine the ear of all rats. We cleaned debris and cerumen layout at the external ear canal. Any rat with external or middle ear infection was excluded from the study.

Thirty animals were divided into three groups of 10 animals each. 1% terbinafine solution was administered to the first group (group T). The second group (group G) was administered 40 mg/ml gentamicin solution (ototoxic control). The third group (group S) was administered saline solution (negative control). 26 animals completed the study without tympanic membrane perforation or any complications after drug application (Table 1). One animal from the terbinafine group was excluded from the study because of ear infection with purulent drainage and three animals from the gentamicin and saline groups died under anesthesia during intratympanic drug application.

**Table 1:** The test groups and solutions

**Group**	**Solution**	**Animals(n=26)**
T	Terbinafine solution	9
G (Ototoxic control group)	Gentamycin solution	8
S (Negative control group)	Saline solution	9

### Study design

The animals were anesthetized with ketamine (100 mg/kg Ketalar; Pfiser Ltd., Vienna, Austria) and xylazine (7.5 mg/kg Rompun; Bayer Ltd., Leverkusen, Germany) given by intraperitoneal injection. Under general anesthesia, after ear microscopic examination, pretreatment DPOAE measurements and ABR testing were performed from the left ears of animals in all groups. After baseline measurements, an anterosuperior myringotomy was performed with a 28-gauge dental needle and then test solutions were applied in the middle ear. The volume of the intratympanic dose was changed between 0.03-0.05 ml according to the volume of the individual rat’s middle ear. The treatment was started after baseline measurements and repeated once every two days for fifteen days. Two weeks after the last application, DPOAE measurements and ABR testing were obtained again and compared with the pretreatment values.

### Hearing assessments

Hearing was assessed by DPOAE and ABR under general anesthesia. All measurements were performed in a quiet room.

#### DPOAE testing

Otodynamics ILO-288 Echoport equipment (Otodynamics Ltd., London,UK) was used to measure DPOAE. Once the probe was placed in external ear canal, the measurements were performed. The sound stimulus that composed DPOAE consisted of two simultaneous permanent pure tones at different frequencies. The stimulus parameters L1 = L2 = 80 dB SPL with a f1/f2 ratio of 1.22 were used and the amplitude of the DPOAE signal was recorded. DPOAE were obtained at seven different frequencies ranging from 1000 to 8000 Hz (1001, 1501, 2002, 3003, 4004, 6006, 7996).

#### ABR testing

The ABR test was done on the left ear and the records were obtained through two channels. Interacustic EP25 instrument and ABR 3A insert earphone were used to evaluate for ABR threshold. Subdermal needle electrodes were used to record the responses. The active electrode was located at the vertex, in the midline of the scalp. The reference electrodes were located in both mastoid regions. The ground electrode was located on the glabella. The ABR test was done by 1000 click stimulus at a rate of 21 times/sc and 100 to 3000 Hz band-pass filters. Measurements were obtained at 70 dBnHL and decreased by increments of 20 dB until the threshold was approached, where 10 dB increments were instituted. Repeatibility was confirmed, and the test was performed twice to determinate threshold. ABR threshold was defined on the fifth wave.

### Statistical analysis

The SPSS statistical software package (SPSS, version 16.0 for windows; SPSS Inc., Chicago, Illinois, USA) was used to perform all statistical calculations. Normal distribution of the variables was tested using the One-Sample Kolmogorov–Smirnov test. Results were expressed as mean ± SD. Student’s t test was used to compare the ABR thresholds and DPOAE values before and after drug administration in each group. A one-way analysis of variance (ANOVA) with the Bonferroni post-hoc test was used to compare the ABR thresholds and DPOAE values between the groups. Differences were accepted statistically significant at a p value < 0.05.

Sample size was calculated according to the results of the first twelve animals in the study (α: 0.05, β value 0.20, study power: 80%). We determined that at least 8 animals were required in each group. We also performed a post hoc power analysis based on posttreatment ABR results (effect size:1, α:0.05) that revealed the study power as 98%.

## Results

**Figure 1:**
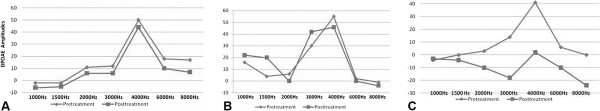
**Graph demonstrating DPOAE amplitudes before and after terbinafine solution (A), saline solution (B) and gentamicin solution (C).** There were no statistically significant differences in DPOAE measurements for terbinafine solution (p > 0.05). There were no statistically significant differences in DPOAE measurements for saline solution (p > 0.05). There were statistically significant differences at 3000, 4000, 6000, and 8000 Hz of DPOAE values for gentamicin solution (p < 0.05).

Pre and post-treatment DPOAE responses for all tested frequencies of group T and Group S showed no statistically significant difference (Figure 1A-1B). However, post-treatment DPOAE responses were found to be lower than pretreatment DPOAE responses in group G and the differences were statistically significant for 3000, 4000, 6000, and 8000 Hz (Figure 1C).

**Figure 2:**
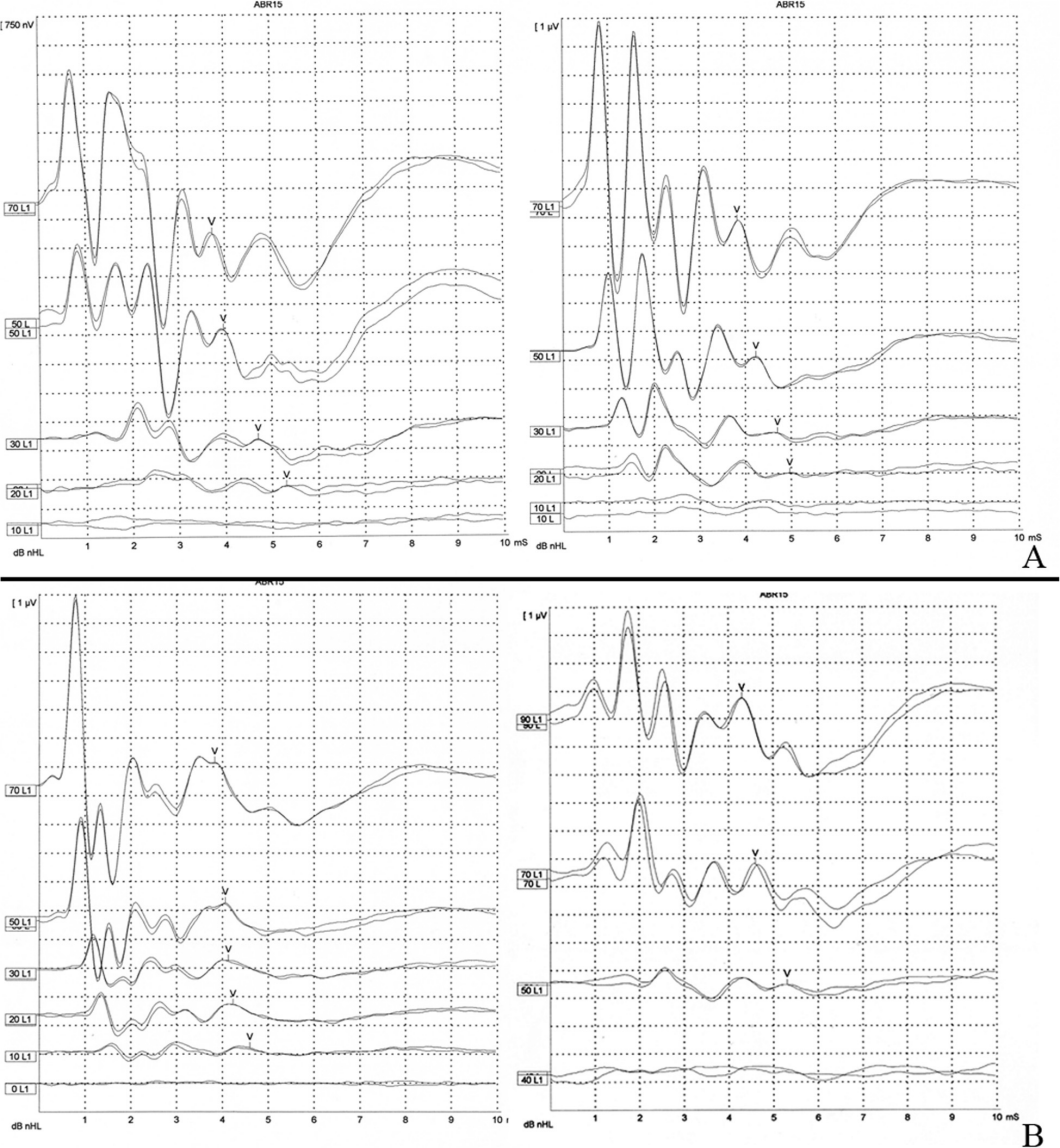
**Graphs demonstrating two examples of ABR thresholds before and after terbinafine solution (A) and gentamicin solution (B).** There were no statistically significant differences in ABR thresholds before and after terbinafine solution (p > 0.05). However; ABR thresholds were significantly increased, after gentamicin application (p < 0.05).

Table 2 demonstrates the ABR thresholds for click stimuli before and after drug administration in each group. Mean ABR thresholds before and after drug application for group T and group S showed no statistically significant difference for group T and group S. However; following the gentamicin application a statistically significant difference in ABR thresholds were shown. Figure 2 demonstrates two examples of ABR recordings before and after terbinafine and gentamicin solution application.

**Table 2:** A**uditory brainstem response thresholds and hearing levels before and after application of various agents**

**Group**	**Pretreatment**	**Posttreatment**	**P**
Terbinafine	21.11±7.81	28.88±13.64	*0.088*
Gentamycin	20.00±5.34	50.00±13.09^+,&^	0.001^*^
Saline	25.55±7.26	27.77±4.40	*0.447*
p (ANOVA)	0.232	0.001	

* Statistical analysis was performed student’s t test p< 0.05.

^+^ p= 0.002 (post hoc Bonferroni) compared with group terbinafine.

^&^p= 0.001 (post hoc Bonferroni) compared with group saline.

When post-treatment ABR thresholds and DPOAE values were compared between the groups, there were statistically significant differences DPOAE values for all frequencies and ABR thresholds. DPOAE values for 3000, 4000, 6000, 8000 Hz in the group G were significantly decreased compared to other groups. ABR thresholds also were significantly increased in the group G compared to other groups (Table 2).

There was no statistically significant difference between pretreatment and post-treatment DPOAE responses and ABR threshold levels for groups T and S. However, the group G demonstrated significant deterioration of ABR thresholds and DPOAE responses, except for at 1000, 1500, and 2000 Hz.

## Discussion

Topical antifungal drugs that are used to treat otomycosis have many positive advantages compared to systemic antifungal drugs. The potential advantages of topical antifungal drugs are local application and the desired higher tissue concentration at the affected site after application [4]. Although there are potential advantages, topical antifungal drugs may lead to a serious risk to the audiovestibular system. Knowing any probable ototoxic properties will guide clinicians in deciding on the most appropriate drugs for treating otomycosis.

The external ear canal may be linked with the middle ear space in many patients with otomycosis. This group includes patients with tympanic membrane perforations, open mastoid cavities and ventilation tubes [5]. When a communication between the external and middle ear space is present, any topical potentially ototoxic agent may diffuse the inner ear through the round window membrane (RWM). Other passage ways are the annular ligament of the oval window, the fistula ante fenestra, microfissures and vascular structures [13]. The RWM represents merely a soft tissue barrier between the middle and inner ears and is accepted as the likely way for ototoxic agents to pass from the middle ear to the inner ear [5,14]. Studies of the RWM demonstrate that its permeability depends on the membrane’s morphological integrity, the molecular weight of the agents, and the presence and duration of inflammation of the middle ear [15,16]. Drug concentration, frequency of exposure and duration are important factors affecting ototoxicity.

Terbinafine solution is an effective treatment modality for mycotic infections of the external ear canal. The mechanism of fungicidal activity of terbinafine is related with particular inhibition of fungal squalene epoxidase that leads to ergosterol deficiency and an accumulation of intracellular squalene [17]. Studies have reported that terbinafine has in vitro effects against Candida, Aspergillus and other pathogenic fungi [7,8]. It is considered effective and safe; however its probable ototoxic effect has been unnoticed, particularly when administered topically in the presence of tympanic membrane perforation, ventilation tubes or an open mastoid cavity.

The present study, we researched the probable ototoxic effect of terbinafine solution on the hearing of rats in which we applied drug for two weeks. The measurements of DPOAE and ABR are commonly used to research ototoxicity. DPOAE is a noninvasive method and provides early diagnosis of cochlear damage caused by topical solutions, which are generally detected first in the outer hair cells [18]. Although a practical method, DPOAE has some limitations. The effusion in the middle ear cavity or the presence of a perforation in the tympanic membrane affects DPOAE responses. Also, the size of tympanic membrane perforation affects DPOAE responses [19]. With this in mind, we did not perforate widely the tympanic membranes of the rats and we applied drugs in the middle ear by intratympanic (IT) injection. IT injection allows a desired much higher concentration of the drugs within the inner ear compared with systemic administration [20]. IT injection also prevents systemic side effects of drugs.

In the present study, after IT application of the terbinafine solution, there was statistically neither a significant decrease of DPOAE amplitudes nor an increase of ABR threshold levels. The posttreatment DPOAE amplitude values were less, but this decrease was not statistically significant (Figure 1A). The posttreatment mean ABR threshold levels were higher than the pretreatment values, but this increase was not statistically significant (Table 2). This condition may be related to the inflammation that was caused by topical administration of the terbinafine in the middle ear cavity. The histopathologic analyses would be beneficial which a wise undertaking as some agents cause diffuse osteoitis and inflammation that may only effect the ABR and DPOAE measurements at an interval longer than two weeks posttreatment. In the group G, we obtained a significant decrease of DPOAE responses and also a significant increase of mean ABR threshold levels, which once again showed the ototoxic effect of the aminoglycoside antibiotics.

We found only one recently published study assessing ototoxic effect of terbinafine in the literature [12]. In that study on ototoxic effect of terbinafine by Aydın et al. hearing was assessed by using ABR alone. In the present study, however, we assessed ototoxicity by using both DPOAE and ABR. Aydın et al. detected ototoxicity by performing ABR seven days after the last administration of terbinafine. However, this ototoxic effect may be temporary and may disappear in the subsequent measurements, as they mentioned as a limitation in the discussion section of their study. We repeated hearing tests two weeks after the last administration of terbinafine. Although we detected a decrease in the amplitudes of DPOAE and an increase in the hearing thresholds of ABR, the results were statistically insignificant.

The present study did not observe any ototoxic effect of terbinafine solution. There are, however, some limitations of our study that we have to mention. First, this study demonstrated only the results of auditory tests for assessment of ototoxicity. We did not perform any vestibular tests for evaluating the vestibular system. Secondly, we could not perform histopathological examination to assess the effects of the terbinafine solution on audiovestibular organs. Further studies that are combined with more definitive electrophysiological, vestibular tests and histopathological examination are needed to assess the effects of terbinafine solution on the audiovestibular system.

## Conclusions

Terbinafine solution is a broad spectrum antifungal agent effective in the treatment of otomycosis. The present study demonstrated that its direct administration in the middle ear of rats does not affect inner ear function as measured by ABR and DPOAE responses. Terbinafine solution should be applied carefully because its safety has not yet been demonstrated in patients who have ventilation tubes or tympanic membrane perforation.

## Competing interests

The authors declare that they have no competing interests.

## Authors’ contributions

MS designed the study, collected datas and wrote manuscript. MAS designed the study, collected datas and wrote manuscript. FK designed the study, collected datas and wrote manuscript. AA collected datas. All authors read and approved the final manuscript.
